# Complement biomarkers in IgA nephropathy: current insights and clinical relevance

**DOI:** 10.3389/fneph.2026.1918450

**Published:** 2026-09-24

**Authors:** Altynay Balmukhanova, Chee Eng Chan, Jonathan Barratt

**Affiliations:** 1 Faculty of Medicine and Healthcare, Al-Farabi Kazakh National University, Almaty, Kazakhstan; 2 Department of Nephrology, Hospital Tengku Ampuan Afzan, Kuantan, Malaysia; 3 Mayer IgA Nephropathy Laboratories, Department of Cardiovascular Sciences, University of Leicester, Leicester, United Kingdom; 4 The John Walls Renal Unit, Leicester General Hospital, Leicester, United Kingdom

**Keywords:** biomarker, complement pathways, IgA nephropathy, precision medicine, target therapy

## Abstract

IgA nephropathy (IgAN) is the most common form of primary glomerulonephritis worldwide and is a major contributor to the global burden of chronic kidney disease. Strong evidence highlights complement activation as a key driver of the disease pathogenesis and its progression. In this regard, the emergence of new therapies targeting the complement system has further increased interest in specific biomarkers that can help in diagnosis, prognosis, and assessment of response to therapy in patients with IgAN. Although complement biomarkers show promising associations with disease severity and prognosis, most remain exploratory and lack adequate validation for routine clinical application. Further research should focus on standardization and prospective multicenter validation of putative biomarkers to facilitate a biomarker-driven approach to the use of complement inhibitors in IgAN. Therefore, this review summarizes a growing body of evidence on the role of complement-related biomarkers in IgAN and discusses their diagnostic and prognostic potential, as well as several limitations for implementation in clinical practice.

## 1 Introduction

IgA nephropathy (IgAN) is the most common biopsy-reported primary glomerulonephritis worldwide (1). Data from the UK RaDaR cohort demonstrate that a substantial proportion of patients with IgAN face a lifetime risk of kidney failure exceeding 75%, underscoring the progressive nature of the disease (2).

The complement system is a key component of innate immunity and a major regulator of the adaptive immune system, and its dysregulation contributes to the development and progression of several glomerular diseases (3–5). Since the pathogenesis of IgAN has become better understood, our insights into the role of complement in its development have also increased significantly (6, 7). The protein components of the cascade are of particular interest as diagnostic and prognostic biomarkers, as they support the development of emerging targeted strategies (6, 8–19).

Given that a one-size-fits-all approach in IgAN is unlikely to be effective, the development and validation of novel biomarkers are increasingly important for patient stratification, treatment selection, and monitoring treatment response. This review summarizes the role of complement biomarkers in IgAN, addressing their diagnostic utility in reflecting disease severity and their prognostic value in predicting therapeutic response and clinical outcomes.

## 2 Complement pathways in IgAN pathogenesis

IgAN is driven by a multi-hit pathogenic process in which complement activation plays a central role in amplifying glomerular injury (6, 20). The disease is initiated by the production of galactose-deficient IgA1 (Gd-IgA1), which forms circulating Gd-IgA1-containing immune complexes (Gd-IgA1-IC) with anti-glycan antibodies (20, 21). These Gd-IgA1-IC deposit in the mesangium and trigger local inflammatory responses, predominantly via activation of the alternative (AP) and lectin (LP) pathways, with limited evidence for classical pathway (CP) involvement (6, 20–24) (**Figure 1**).

**Figure 1:**
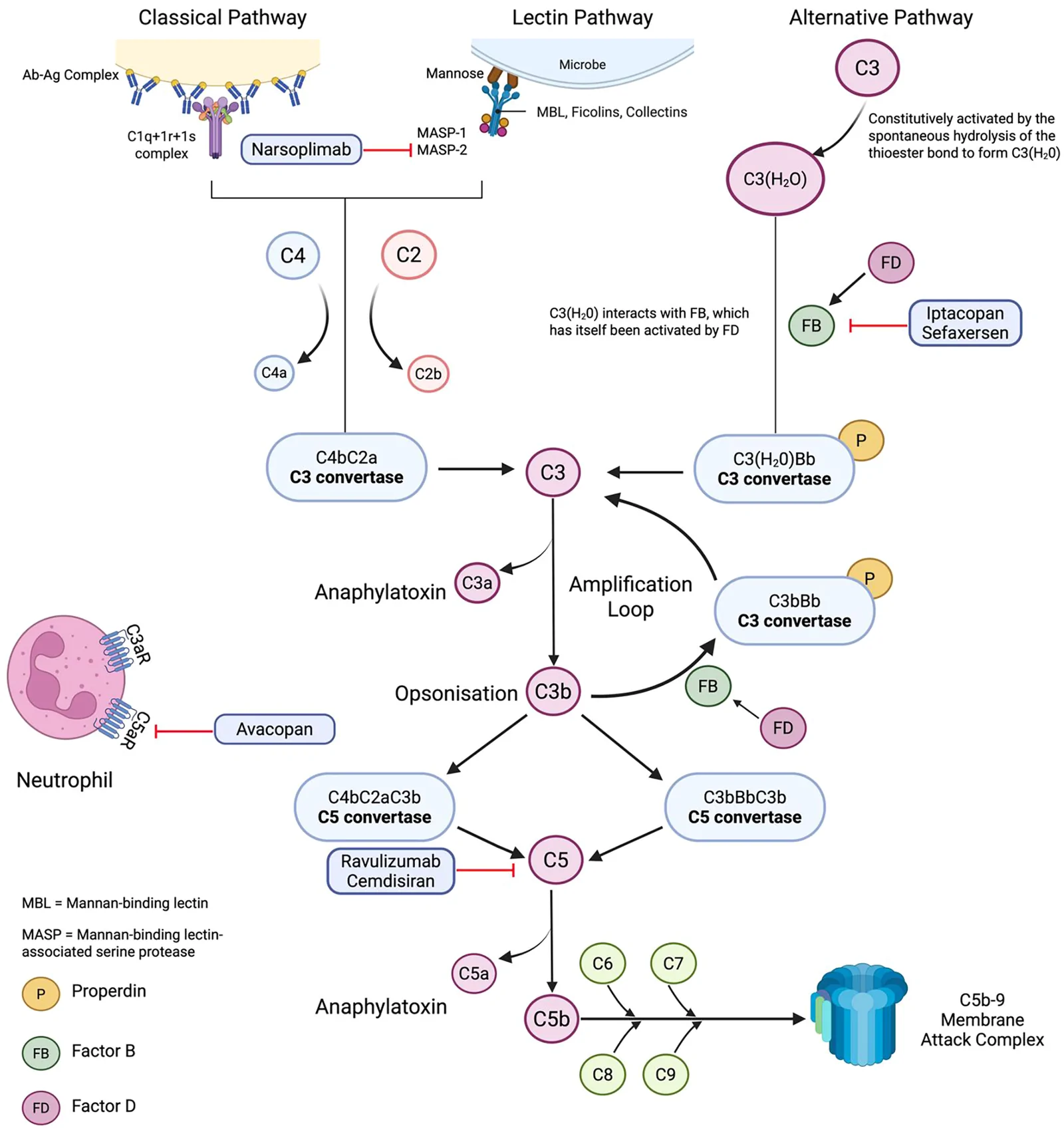
The complement pathway cascade. Created with BioRender.com.

The AP is considered a dominant driver of complement activation in IgAN (4, 7, 8). It is constantly active at low levels (4, 22) and may be enhanced by Gd-IgA1-IC deposited within the mesangium (6, 23). Moreover, the AP contributes substantially to inflammation and tissue injury and amplifies the consequences of CP and LP activation (7). The LP, increasingly recognized as an important contributor to glomerular injury in IgAN, alongside the AP (24), is mediated by carbohydrate-recognition molecules such as mannan-binding lectin (MBL) and ficolins, which bind to Gd-IgA1-IC (23, 25, 26). This triggers downstream activation of MBL-associated serine protease-2 (MASP)-2 and subsequent cleavage of complement components, contributing to inflammation (26).

Each pathway converges at the level of C3 activation, leading to the generation of the terminal pathway (TP), the formation of pro-inflammatory mediators including C3a, C5a, and the membrane attack complex (C5b-9). C3a and C5a recruit inflammatory cells and promote cytokine release (6, 27, 28), whereas C5b-9 induces mesangial cell proliferation, oxidative stress, and extracellular matrix expansion, ultimately contributing to progressive kidney injury (27, 28).

Overall, persistent complement activation not only promotes mesangial inflammation but may also contribute to endothelial damage, primarily associated with the involvement of the AP (29). Several studies have found histological evidence of acute or chronic thrombotic microangiopathy (TMA) in a subset of patients with IgAN, particularly in severe cases, suggesting that endothelial damage may represent an additional mechanism of kidney injury and thus underlie the clinical heterogeneity and diverse phenotypes of the disease (30–33).

## 3 Complement biomarker landscape in IgAN

A broad range of biomarkers, including complement-related ones, have been studied in IgAN, reflecting the disease heterogeneity and the complexity of its underlying mechanisms. These biomarkers span multiple biological compartments, including renal tissue, blood and urine, and provide insights into various aspects of disease activity (34–36). Key complement-related biomarkers in IgAN are summarized in **Table 1** (37–60).

**Table 1:** Complement-related biomarker landscape in IgA nephropathy.

Biomarker	Pathway	Sample	Key findings	References
FHR-1, FHR-1/fH	Alternative	Serum	Elevated in IgAN, even with normal eGFR, is associated with progression, suggesting potential prognostic value	Medjeral-Thomas et al, 2017 (37) Tortajada, et al, 2017 (38)
FHR-5, FHR-5/fH	Alternative	Serum	Associated with more severe clinical and histological features and may predict treatment response	Medjeral-Thomas et al, 2017 (37) Zhu et al, 2018 (39);
Bb	Alternative	Serum	Associated with baseline histopathologic vascular and tubular lesions and with worse long-term renal outcomes	Ștefan et al., 2023 (40)
Ba	Alternative	Plasma	Elevated in IgAN; it correlates with disease activity, decreased after immunosuppression	Chiu et al., 2021 (41)
FH	Alternative	Urine	Associated with proteinuria and disease severity	Onda et al, 2011 (42)
Mesangial C4d	Lectin	Tissue	Consistently associated with severe histological lesions and worse renal outcomes, it is a well-established marker of intrarenal complement activation	Espinosa et al, 2014 (43) Jiang et al, 2021 (44)
C4d	Lectin	Urine	Associated with proteinuria and progression risk, a promising non-invasive marker	Wang Z et al., 2022 (45) Dong et al., 2024 (46)
MBL	Lectin	Urine	Correlated with proteinuria, eGFR decline, hypertension, and more severe histological lesions	Wang Z et al., 2021 (47)
MASP-3	Lectin	Serum	Lower levels observed in progressive IgAN	Medjeral-Thomas et al, 2018 (48)
C4	Classical/ Lectin	Serum	Correlated with proteinuria and eGFR decline, morphological lesions; it was shown as an independent risk factor for disease progression	Zhu et al., 2015 (49) Bi et al., 2019 (50)
sC5b-9	Terminal	Urine	Correlated with kidney inflammation and progression of glomerulosclerosis, proteinuria	Onda et al, 2011 (42) Medjeral-Thomas et al, 2018 (48) Yu et al., 2022 (51)
Complement panel (u-PTX-3, u-MBL, u-C4c)	Mixed	Urine	Associated with proliferative lesions (u-PTX-3, u-MBL) and chronic damage (u-C4c); promising panel for disease phenotyping	Nurmi et al., 2024 (52)
C3, C3/C4 ratio, IgA/C3 ratio	Overall/ non-specific	Serum	Lower C3 associated with worse prognosis; lower C3/C4 linked to worse clinical and pathological features; IgA/C3 ratio linked to diagnosis and progression in some cohorts	Tomino et al., 2000 (53) Tringali et al., 2024 (54) Pan et al., 2018 (55) Ergun et al., 2026 (56) Gong et al., 2019 (57) Sirali et al., 2023 (58) Torikoshi et al., 2024 (59) Swamy A, 2025 (60)

Currently, no validated markers are available in routine clinical practice beyond estimated glomerular filtration rate (eGFR) and proteinuria, which are non-specific for IgAN and may indicate the general progression of chronic kidney disease (CKD) due to nephron loss (61). Most complement biomarkers remain exploratory and have been primarily evaluated in single center, mechanistic or translational studies, although more recently data from large global phase 3 clinical trials are begin to be reported (6, 61). Nevertheless, considering that these biomarkers may reflect activation of specific complement pathways, they hold considerable promise for improving disease stratification and supporting pathway-targeted therapeutic strategies.

### 3.1 Tissue (histological) complement biomarkers

Mesangial deposition of MBL, L-ficolin, MASPs, C4d and other LP components is observed in approximately 25–50% of IgAN biopsies, usually in the absence of C1q, supporting LP involvement (24, 43, 62).

C4d is one of the most extensively studied and clinically relevant LP biomarkers in IgAN, detectable in tissue, serum and urine. A meta-analysis of 12 studies with 1,251 participants reported an overall prevalence of C4d deposits was 34% (95% CI, 27%-41%) (44). Multiple studies have demonstrated the prognostic significance of C4d staining, as its deposition is associated with greater proteinuria and lower eGFR, both of which are associated with CKD progression. In fact, the presence of C4d in renal tissue has been reported as an independent predictor of progression in IgAN (43, 62–66). In addition, C4d deposition has also been linked with adverse Oxford MEST lesions, including mesangial hypercellularity and segmental sclerosis (33, 44).

Notably, similar patterns of C4d staining in IgAN have been reported independently by multiple investigators, making it a promising biomarker of intrarenal LP activation (43, 44). However, despite its prognostic value, C4d has not yet been incorporated into standard risk-stratification models and clinical decision-making (44). Furthermore, since C4d is associated with the LP, and LP activation is only seen in a subset of IgAN patients, it is unclear how relevant it is to the global IgAN patient population (23, 24, 62, 63).

In more than 90% of cases, mesangial IgA and C3 deposits are observed in the kidney biopsy. The absence of C3 deposits is associated with a more favorable disease course (54, 66, 67). *In situ* detection of convertases (C3bBb, C4b2a) indicates ongoing glomerular complement activation in most C3-positive IgAN biopsies; the presence of C3bBb has been correlated with endocapillary proliferation and focal segmental glomerulosclerosis, C5b-9 deposition, and worse renal outcomes, supporting a pathogenic role for AP activation (67). However, studies of mesangial C3 and C5b-9 have failed to show consistent prognostic value in IgAN, and this likely reflects the fact that studies have measured different C3 fragments (47, 66–68). C3c staining measures a short-lived C3 fragment indicating ongoing complement activation, while staining for C3d identifies a C3 fragment covalently bound to cell membranes and can be associated with complement activation that may have occurred a number of months prior to the kidney biopsy (23, 66, 69). Similarly, staining for C5b-9 reflects complement activation that may have happened months earlier, limiting its usefulness in assessing present complement activation (23, 67, 69).

### 3.2 Circulating complement biomarkers

Several circulating biomarkers associated with distinct complement pathways have been investigated. Among these, factor H-related proteins, a family of human plasma proteins (FHR-1–FHR-5) encoded by the CFH gene locus, are of particular interest for their role in regulating the AP (38, 39, 70). FHR proteins may compete with factor H (fH), the major negative regulator of complement C3 activation. As reported by Medjeral-Thomas NR et al., plasma FHR-1 levels were elevated in IgAN patients, even in those with normal renal function; however, FHR-1 levels were negatively correlated with eGFR (37). At the same time, the plasma fH level did not differ between IgAN patients and healthy subjects (38). Considering this, along with FHR-1, it has been proposed that the FHR-1/fH ratio would better reflect the balance between complement control and its dysregulation, and, as shown in the study by Tortajada A et al, the ratio is significantly elevated in IgAN patients (38). Moreover, higher levels were associated with a progressive rather than stable disease course (47).

Another member of the FHR family, FHR-5, has been reported to be elevated in IgAN, but levels showed no significant correlation with eGFR (37). In contrast to FHR-1, neither FHR-5 nor the FHR-5/fH ratio was associated with disease progression (37). Furthermore, the FHR-5/fH ratio did not differ significantly between IgAN patients and healthy subjects (37). Nevertheless, higher serum FHR-5 levels were associated with more pronounced histological changes, as measured using the Oxford MEST score, and have been associated with a reduced response to immunosuppressive therapy (37, 39, 48). These findings indicate that the FHR-5 may be more useful for predicting and monitoring treatment response than for assessing disease progression. In addition, the higher FHR-5 levels showed a weak but significant correlation with proteinuria and were higher in IgAN patients with hypertension (39).

Other noteworthy biomarkers reflecting activation of the AP are the cleavage products of complement factor B – Bb and Ba, which are detectable in the circulation. It should be noted that for accurate detection of these activation fragments blood must be collected in EDTA to minimize *ex vivo* complement activation which occurs during clotting (40). Of these, Bb has been more extensively studied in IgAN. Higher Bb levels were associated with baseline histopathologic vascular and tubular lesions and with worse long-term renal outcomes, with the authors suggesting a role for AP activation at the endothelial surface (40). Plasma Ba levels have also been reported to be elevated in IgAN patients compared to controls and patients with focal segmental glomerulosclerosis; and to correlate with disease activity and decrease after immunosuppression (41).

MASPs are the key serine proteases of LP activation (6, 24). Among the 3 proteases in the MASP family, MASP-3 has been shown to be associated with disease severity and progression. In a cohort of IgAN patients, plasma MASP-3 levels were lower than in healthy subjects and were the lowest in patients with advanced disease. Furthermore, low MASP-3 levels were associated with increased deposition of C3, C3d, C5b-9, and FHR5 in the glomeruli, and with more severe histological lesions (48). Similar findings were reported in a Swedish study, where decreased plasma MASP-3 levels were consistently associated with lower eGFR and with endocapillary proliferation and crescents (52). These data support the potential role of MASP-3 as a circulating biomarker of IgAN severity and progression risk and suggest it could be considered as a future component of a complement biomarker panel.

Data on the value of serum C4 levels as a useful biomarker in IgAN are limited. In 2014, Zhu et al. found that patients with lower C4 levels at diagnosis tended to have poorer outcomes over the follow-up period, even though these patients had less extensive lesions at the time of biopsy (49). A more recent study involving 1,356 Chinese IgAN patients found that serum C4 levels were positively correlated with proteinuria and negatively with eGFR. Serum C4 levels also positively correlated with the extent of tubulointerstitial damage, glomerulosclerosis, and number of crescents, as measured using the MEST-C classification. Additionally, elevated C4 levels were shown to be an independent risk factor for disease progression (50). Several studies have reported that a lower serum C3/C4 ratio was linked to worse clinical and pathological features (55, 56) and that the predictive ability of the C3/C4 ratio was significantly greater than that of either serum C3 or C4 concentration alone (55). Tringali et al. reported that in addition to proteinuria and eGFR serum C3 and C4 levels were associated with risk of progression (54). Furthermore, their addition to both existing prognostic models, the CKD progression model and the International IgAN Risk Prediction Tool, improved predictive performance (54).

A number of studies, particularly in Asian IgAN cohorts, have reported that a high IgA/C3 ratio is an independent predictor of worse kidney survival, alongside proteinuria, hypertension, and baseline renal function in IgAN (57, 59, 68). These associations are reported to be specific to IgAN compared to other glomerular diseases (53, 57, 60, 71), and have been associated with both histologic severity (47) and risk of disease progression (59, 60, 68). It has been proposed that the IgA/C3 ratio should be included alongside clinical data and biopsy findings in Asian patients when assessing risk of progression (57, 59). However, the implications of existing data on IgA/C3 ratio are unclear in non-Asian populations due to inconsistent results from cohort studies (57, 60, 68).

### 3.3 Urinary complement biomarkers

Urinary complement biomarkers in IgAN are proposed as noninvasive indicators of intrarenal inflammation, particularly reflecting AP and LP activation. These biomarkers have emerged as potential tools for assessing disease activity and prognosis in IgAN.

Elevated urinary C4d levels have been associated with greater proteinuria, more severe histopathological injury, and an increased risk of kidney disease progression, independent of conventional clinical and pathological risk factors. In addition, higher urinary C4d levels have been associated with poorer response to immunosuppressive therapy, mainly steroids, suggesting a potential role in treatment monitoring (46). Similarly, urinary factor H and soluble C5b-9 (sC5b-9) levels have been correlated with proteinuria, kidney function decline (51), interstitial fibrosis, and global glomerulosclerosis, whereas urinary properdin correlated primarily with proteinuria only (42).

A study by Wang et al., including 100 IgAN patients with varying degrees of glomerular crescent formation, demonstrated that urinary C3a and C5a (common pathway), C4d and MBL (LP), and C5b-9 (TP) were positively correlated with the proportion of glomerular crescents, serum creatinine, and proteinuria (47).

In a Swedish cohort study of patients with IgAN and IgA vasculitis nephritis, urinary pentraxin 3 (PTX-3) and MBL were associated with active proliferative kidney lesions, whereas higher urinary C4c levels correlated with chronic fibrosis and more rapid kidney function decline (52). These findings support the concept that urinary complement excretion likely reflects intrarenal complement activation. However, the clinical applicability of these biomarkers remains limited. Most studies lack disease control groups with comparable proteinuria, raising the possibility that increased urinary complement levels may, at least in part, reflect nonspecific leakage across a damaged glomerular filtration barrier rather than disease-specific complement activation.

Nonetheless, in the context of emerging complement-targeted therapies, urinary complement components, particularly C4d and soluble C5b-9, may hold promise as pharmacodynamic or stratification biomarkers, pending further validation in well-controlled cohorts.

### 3.4 From biomarkers to targeted therapy

Complement activation has emerged as a central mechanism in IgAN pathogenesis, driving interest in complement biomarkers as prognostic, mechanistic, and pharmacodynamic tools, as well as the development of complement-targeted therapies. Studies have implicated the AP, LP, and TP through findings such as mesangial deposition of C3 and C4d, elevated plasma Bb, increased urinary sC5b-9, and abnormalities in serum complement proteins. These biomarkers have consistently been associated with greater proteinuria, more severe histologic injury, and worse kidney outcomes (9).

The strongest clinical evidence to date has come from studies targeting the AP. Iptacopan, an oral factor B inhibitor, has been shown to significantly reduce proteinuria and the rate of loss of kidney function with associated sustained modulation of blood and urine complement biomarkers in both phase 2 and phase 3 studies (11–14). Iptacopan suppressed AP activity, as measured with the Wieslab assay, increased serum C3 levels and reduced urinary sC5b-9, supporting target engagement and pharmacodynamic activity (12, 14). The final 24-month APPLAUSE-IgAN data demonstrated that iptacopan treatment, compared to placebo, resulted in an annualized total eGFR slope of -3.10 vs. -6.12 ml/min/1.73 m²/year (difference 3.02 ml/min/1.73 m²/year; 95% CI, 2.02 to 4.01; adjusted P < 0.001) and a composite kidney-failure endpoint hazard ratio of 0.57 (95% CI 0.40-0.81; adjusted P = 0.003) (14). These data have supported an initial accelerated approval (2024) and now a traditional approval (2026) from the US Food and Drug Administration (FDA) for iptacopan for the treatment of patients with primary IgAN at risk of progressive loss of kidney function (72–74).

Sefaxersen (formerly IONIS-FB-LRx; RO7434656) is a GalNAc-conjugated antisense oligonucleotide that selectively inhibits hepatic synthesis of complement factor B, thereby suppressing activation of the AP (15). By reducing circulating factor B levels, sefaxersen decreases formation of the AP C3 convertase (C3bBb), limiting complement amplification while preserving the CP and LP. In a Phase 2 open-label trial including patients with IgAN and persistent proteinuria despite optimized supportive care, treatment with monthly subcutaneous sefaxersen for 24 weeks resulted in substantial reductions in plasma factor B, Bb, and urinary Ba levels, confirming effective pharmacodynamic inhibition of the AP. These biomarker changes were accompanied by an approximately 43% reduction in proteinuria and stable kidney function, with an overall favorable safety profile and no significant increase in serious infections (15). Sefaxersen is now being evaluated in the Phase 3 IMAGINATION trial (16).

LP inhibition has also been explored extensively. Narsoplimab, a monoclonal antibody targeting MASP-2, showed early promise in reducing proteinuria and stabilizing kidney function in phase 2 study, supporting the pathogenic role of LP activation in IgAN (10). However, the subsequent phase 3 ARTEMIS-IgAN program was discontinued after failing to demonstrate sufficient efficacy, highlighting the heterogeneity of complement activation in IgAN and the potential need for biomarker-guided patient selection (75).

Therapies targeting the TP have likewise entered clinical investigation. Ravulizumab is a long-acting monoclonal antibody that inhibits complement C5, thereby preventing the generation of C5a and formation of the membrane attack complex. In a Phase 2 randomized, double-blind, placebo-controlled trial, ravulizumab significantly reduced proteinuria by 30.1% compared with placebo at Week 26, with sustained reductions through Week 50 while maintaining a favorable safety profile (17). The obtained results confirmed the fundamental possibility of inhibition of TP in IgAN. A prespecified interim analysis demonstrated a statistically significant and clinically meaningful reduction in proteinuria at Week 34, with the final analysis evaluating long-term kidney function at Week 106 (76).

Cemdisiran is a small interfering RNA (siRNA) therapeutic that selectively suppresses hepatic synthesis of complement component C5, thereby inhibiting generation of C5a and formation of the membrane attack complex. In a Phase 2 randomized, double-blind, placebo-controlled trial, cemdisiran significantly reduced proteinuria, with a placebo-adjusted 37.4% reduction in 24-hour urine protein-to-creatinine ratio at Week 32, accompanied by near-complete suppression of circulating C5 levels and a favorable safety profile (18). These findings further support the role of terminal complement inhibition in IgAN. However, despite these encouraging Phase 2 results, cemdisiran has not advanced to a Phase 3 clinical trial in IgAN.

Avacopan, an oral selective C5a receptor 1 inhibitor, has been investigated as a strategy to attenuate C5a-mediated inflammation in IgAN. In a small open-label pilot study, avacopan was associated with reductions in proteinuria in some patients; however, the small sample size and uncontrolled study design limit conclusions regarding its efficacy (19). More recently, the retraction of the Phase III ADVOCATE trial publication (77), regulatory concerns regarding the integrity of the supporting clinical data, the FDA proposal to withdraw approval for ANCA-associated vasculitis (78), and reports of serious hepatotoxicity have led to uncertainty regarding the clinical development of avacopan, and its future role in IgAN remains unclear.

Collectively, these trials have established complement inhibition as one of the most rapidly evolving therapeutic areas in IgAN and have highlighted the growing role of complement biomarkers in understanding disease mechanisms, identifying therapeutic targets, and monitoring biological response to treatment.

## 4 Discussion

### 4.1 Challenges and limitations

Although an increasing number of complement-related biomarkers have been described in IgAN, their current clinical applicability remains uncertain (35, 61). The KDIGO 2025 Clinical Practice Guideline states that, apart from proteinuria and eGFR, there are no validated prognostic serum or urine biomarkers for IgAN. At the same time, KDIGO identifies biomarker discovery and validation as a high-priority research recommendation, acknowledging their potential to refine risk prediction and support precision medicine (61). However, bridging this gap between promising research findings and clinical implementation will require overcoming several important challenges.

One of the main challenges is the heterogeneity of IgAN itself, since complement activation occurs through multiple, partially overlapping pathways that differ among patients and may wax and wane during the natural history of the disease (23, 27). Additionally, complement activation in IgAN may show population-specific differences according to ethnicity and histological phenotype (23, 47, 79).

Furthermore, the current body of evidence remains confounded by considerable methodological differences in measuring complement biomarkers across published studies, many of which have been conducted in single-center cohorts, have differed substantially in study design and have used heterogeneous definitions of clinical outcomes (37–39, 45–47, 52–54, 60, 64–66, 68). Differences in assay methodologies and non-standardized cut-off values further reduce reproducibility and external validation across populations (38, 39, 45–47, 52, 53, 57–60, 71). As a result, diagnostic and prognostic performance is moderate and inconsistent, and does not closely align with the characteristics of an optimal biomarker that could be widely used in clinical practice (35, 61). This issue is also relevant to tissue biomarkers, despite their consistently reported associations with adverse renal outcomes (44, 60). At present, reported circulating and urinary biomarkers remain exploratory and need to be validated in prospective multicenter studies (35, 36, 61).

Another important consideration is that each biomarker has its own biological and clinical limitations. Although tissue biomarkers provide the most direct evidence of intrarenal complement activation (23, 43), kidney biopsy, an invasive procedure that may be inaccessible and/or unaffordable in some countries, is unlikely to be a practical approach to repeated assessment of response to treatment (35, 61). Conversely, while circulating biomarkers are more readily accessible, they likely reflect systemic rather than kidney-specific complement activation, potentially limiting their sensitivity to detect complement-mediated kidney injury (23, 35, 36). Urinary biomarkers represent a promising non-invasive approach for monitoring ongoing complement-mediated kidney injury and may better reflect disease dynamics; however, validated predictive urinary complement biomarkers remain scarce (35, 46, 52, 54, 61, 80). Taken together, these findings suggest that no single biomarker approach is likely to capture the complexity of complement activation in IgAN fully.

### 4.2 Future directions

The next stage of development in this field should be not just the identification of new biomarkers, but their clinical validation for patient stratification and selection of targeted therapy. Ideally, treatment selection in IgAN should be based on mechanism of action specific biomarkers that reflect pathophysiological activity requiring suppression. Whereas previous studies focused on proving the role of complement in the pathogenesis of IgAN, the efficacy of complement inhibitors has now been convincingly established in large-scale clinical trials and the priority should now shift towards identifying those patients most likely to benefit from complement inhibition, and which level of the complement cascade should be targeted in individual patients.

Consideration of the clinical heterogeneity of IgAN and its various phenotypes is essential when selecting an appropriate treatment strategy. Thus, complement-mediated endothelial injury and associated microangiopathic lesions may represent a clinically relevant phenotype of IgAN in which complement inhibition could potentially have additional therapeutic value. However, whether patients with IgAN-associated TMA derive preferential benefit from complement-targeted therapies remains unknown, as no trials have specifically evaluated complement inhibition in this subset of IgAN patients. Evidence is currently limited to case reports and this specific aspect of complement inhibition should be a focus of future studies.

The principle of “right pathway, right drug, right patient, right time” emphasizes the importance of biomarker-guided therapy and individualized risk stratification and treatment selection to optimize outcomes. Identifying the dominant pathogenic pathway in each patient at any given time enables clinicians to select therapies more effectively, improve outcomes, minimize unnecessary exposure to immunosuppression, and reduce treatment-related adverse effects. This may allow matching of pathway activity, such as mucosal immune activation, BAFF and/or APRIL signaling, and complement activation, to the most appropriate targeted therapy or combination of therapies. Future validated biomarker panels will support better treatment selection and facilitate the design of ongoing and future clinical trials aimed at individualized treatment approaches. However, current evidence remains limited and largely exploratory. Large, multiethnic, longitudinal studies are required to confirm the validity and practical applicability of the complement biomarkers discussed in this review before they can be routinely implemented in clinical practice.

## 5 Conclusion

Complement biomarkers are a relatively new and rapidly evolving area in IgAN and may serve as a potential bridge between pathogenesis and targeted therapeutic strategies. Tissue, circulating and urinary biomarkers provide complementary insights into disease activity and pathway-specific injury. At the same time, new complement-targeted therapies further highlight the clinical significance of these pathways and the importance of biomarker-based patient stratification to identify those most likely to benefit from targeting the complement system. Continued integration of biomarker research with therapeutic development will hopefully lead to more precise, pathogenetically based treatment strategies in IgAN.
